# Risky decision-making in dementia: Exploring neural correlates and related clinical symptoms

**DOI:** 10.3758/s13415-025-01291-3

**Published:** 2025-04-11

**Authors:** Molly-Eve Day, David Foxe, Grace Wei, James Burrell, Olivier Piguet, Fiona Kumfor, Stephanie Wong

**Affiliations:** 1 College of Education, Psychology and Social Work, Flinders University, Bedford Park, South Australia 5042 Australia; 2 School of Psychology, University of Sydney, Sydney, Australia; 3 Brain and Mind Centre, University of Sydney, Sydney, Australia

**Keywords:** Behavioural-variant frontotemporal dementia, Alzheimer’s disease, Balloon Analogue Risk Task, Reward learning, Voxel-based morphometry

## Abstract

**Background:**

Appropriately balancing potential risks versus rewards is important for affective decision-making in everyday life. Impaired affective decision-making on risk-taking tasks has been reported in individuals with dementia, but the neural correlates of such deficits, and whether they relate to neuropsychiatric symptoms, such as disinhibition and apathy, have not been directly examined.

**Methods:**

Forty-one behavioural-variant frontotemporal dementia (bvFTD), 28 Alzheimer’s disease (AD) patients and 42 healthy controls completed the Balloon Analogue Risk Task (BART), which assessed their ability to weigh risks versus rewards to maximise monetary earnings. Informant-reported measures of disinhibition and apathy were completed. All participants underwent structural magnetic resonance imaging brain scans.

**Results:**

While bvFTD and AD patients showed some impairments on the BART relative to controls, a high degree of variability was observed within patient groups. Poorer BART performance was associated with bilateral medial prefrontal and orbitofrontal cortex atrophy. A hierarchical cluster analysis revealed four groups of patients, with distinct patterns of BART performance, varying levels of disinhibition and apathy, and divergent patterns of brain atrophy. The group that showed the worst performance on the BART (i.e., collected the least money and popped the most balloons) showed the greatest disinhibition and orbitofrontal cortex atrophy.

**Conclusions:**

Our findings highlight the heterogeneous nature of affective decision-making deficits in dementia and uncover important links between BART performance, symptoms of disinhibition and apathy, and orbitofrontal cortex atrophy. Greater understanding of these symptom profiles and underlying neurocognitive mechanisms may help to inform potential management strategies for impaired affective decision-making in dementia.

**Supplementary Information:**

The online version contains supplementary material available at 10.3758/s13415-025-01291-3.

## Introduction

**Fig. 1:**
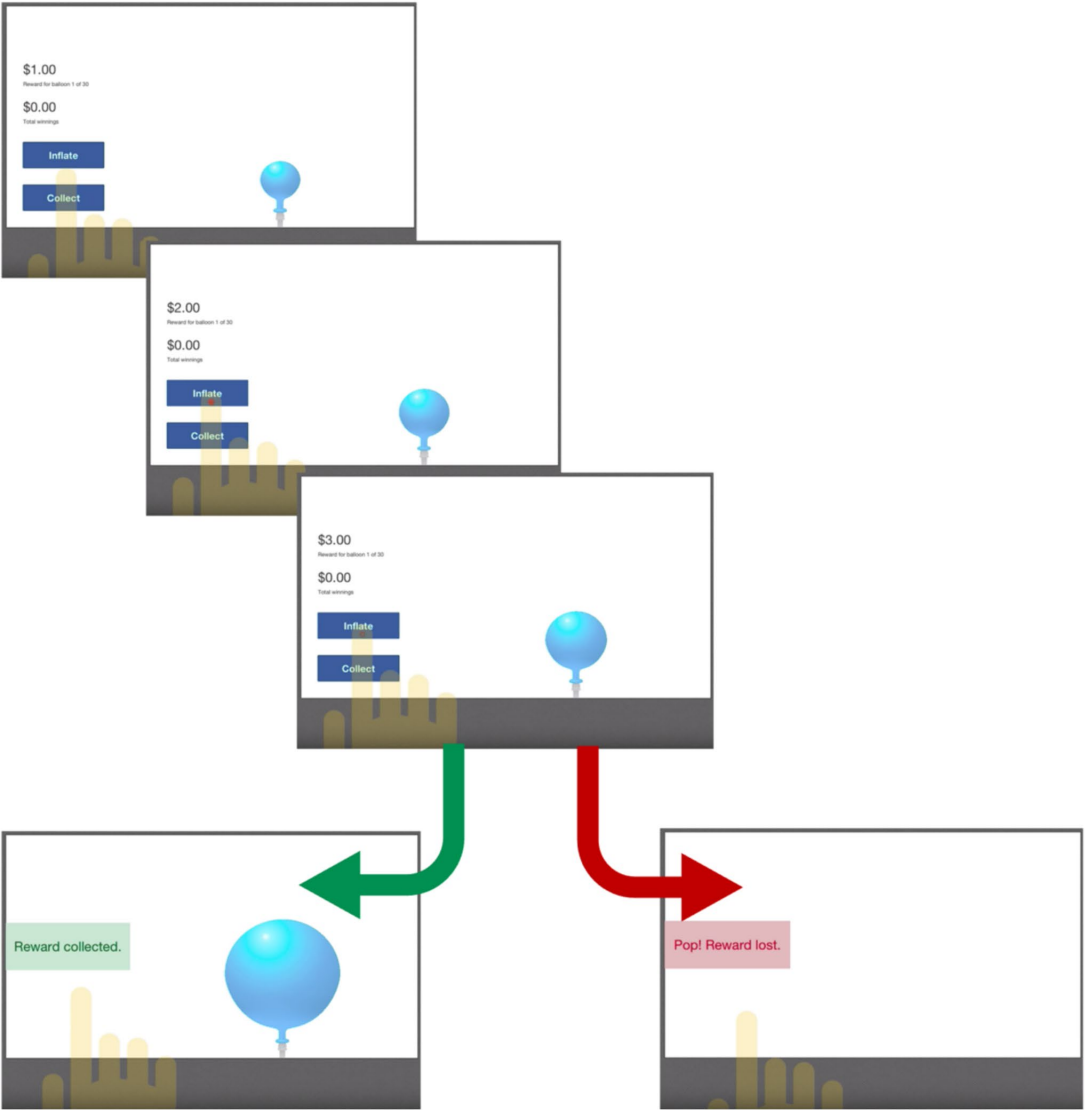
Examples of trial stimuli on the BART, with two possible outcomes following the “inflate” responses: “Reward collected” or “Pop! Reward lost.”

Affective decision-making refers to decisions that are influenced by emotions and reward value. These decisions often involve an element of risk or uncertainty. Although taking risks can sometimes be beneficial (e.g., asking a friend out on a date), excessive risk-taking can lead to maladaptive decision-making. In laboratory settings, the Balloon Analogue Risk Task (BART) (Lejuez et al., 2002) has been used to investigate affective decision-making. On this computerised task, participants choose to either pump a balloon for the opportunity to gain greater rewards (typically monetary) or collect their current amount of reward. Each pump is associated with an increased risk of the balloon popping, which results in loss of the accumulated reward for that balloon (Fig. 1). The BART therefore assesses the ability to weigh risk (potential loss of reward due to the balloon popping) versus reward (potentially greater reward with each pump of the balloon).

To date, the BART has primarily been used in studies of adolescents, which consistently demonstrate that those with higher mean adjusted number of pumps (mean number of pumps per trial/balloon excluding trials where the balloon popped) show higher scores on self-reported measures of impulsivity, sensation-seeking, and disinhibition (Hopko et al., 2006; Lejuez et al., 2002, 2003; White et al., 2008). Aside from research in adolescents, few studies have used the BART to investigate affective decision-making in other relevant clinical populations, such as dementia.

Deficits in affective decision-making have been reported on related delay-discounting and gambling tasks in patients with behavioural-variant frontotemporal dementia (bvFTD) (Bertoux et al., 2015). Behavioural-variant frontotemporal dementia is characterised by marked and progressive declines in personality and social behaviour (Rascovsky et al., 2011), alongside early atrophy of the orbitofrontal cortex (OFC), anterior cingulate, and frontal insula (Rosen et al., 2002). Patients with bvFTD typically present with striking affective deficits, including emotional blunting or reduced emotional responsivity (also known as emotional apathy), impaired emotion recognition and loss of empathy, which have been attributed to orbito-medial prefrontal and insular atrophy (Dermody et al., 2016; Kumfor et al., 2017; Wong et al., 2023). Impaired performance on affective decision-making tasks has also been documented in patients with Alzheimer’s disease (AD) (Bayard et al., 2014), who primarily present with progressive episodic memory deficits and early atrophy in the entorhinal cortex and hippocampus (McKhann et al., 2011). In contrast to patients with bvFTD, AD patients tend to show less emotional apathy (Wei et al., 2019), as well as relatively intact emotion recognition and empathy (Dermody et al., 2016; Kumfor et al., 2017), particularly in the earlier disease stages. Importantly, previous studies identifying affective decision-making deficits in bvFTD and AD (Bayard et al., 2014; Bertoux et al., 2015) differentially attribute these impairments to symptoms of disinhibition (an inability to control inappropriate behaviour or impulses) in bvFTD versus generalised apathy (a loss of motivation and goal-directed behaviour) in AD. Symptoms of disinhibition and apathy have been reported in both bvFTD and AD (Mariano et al., 2020; Wei et al., 2019), although the severity of these symptoms can vary widely both between and within dementia subtypes (Lansdall et al., 2017). It remains unclear whether declines in affective decision-making in these two dementia groups are specifically related to disinhibition or apathy—or both neuropsychiatric symptoms. Furthermore, few studies have explored this issue using an affective decision-making task that incorporates an element of risk, such as the BART.

To the best of our knowledge, only one study to date has examined BART performance in bvFTD. Strenziok et al. (2011) found that bvFTD patients showed lower mean adjusted pumps compared with controls and “collected” their rewards sooner, thus limiting their overall winnings. The authors reasoned that this may have been due to poorer learning over time, such that over the 30 trials, bvFTD patients showed limited learning to increase the number of pumps, in order to maximise their earnings. Given that bvFTD patients typically present with high levels of disinhibition (Rascovsky et al., 2011), these results seem to contradict previous findings in adolescents, which demonstrated that higher mean adjusted pumps on the BART were associated with greater self-reported disinhibition (Hopko et al., 2006; Lejuez et al., 2002, 2003). Using voxel-based morphometry, Strenziok et al. (2011) also demonstrated a link between impaired learning on the BART and right lateral OFC atrophy, suggesting that this region plays an important role in the ability to learn optimal decision-making strategies when balancing risks versus rewards.

While the Strenziok et al. (2011) study revealed important differences in risk-related affective decision-making in bvFTD compared with controls, several limitations remain to be addressed. First, the study did not include a measure of disinhibition, so it is unclear whether BART performance was associated with symptoms of disinhibition. Second, the study did not consider the potential influence of other variables, such as apathy. Of relevance, presence of apathy has been linked with impairments in affective decision-making in both AD and bvFTD (Bayard et al., 2014; Jurgelis et al., 2021; Wong et al., 2023). Furthermore, because Strenziok et al. (2011) only focused on the mean adjusted number of pumps (which excludes the trials on which balloons were popped) as the main outcome variable, behaviour on these excluded trials may not be adequately captured. It is possible that additional variables, such as the number of balloons popped, the mean reaction time per trial, and the total amount of money collected, may provide further insight into affective decision-making processes on the BART. For example, patients with greater disinhibition may pop more balloons, respond faster, and collect less money on the BART. Finally, because the study did not include a dementia comparison group, it remains unclear whether these findings are specific to bvFTD and the patterns of atrophy seen in this dementia group.

The overarching objective of this study was to compare performance on the BART and its clinical and neural correlates in bvFTD and AD patients, as well as healthy controls. In doing so, we aimed to explore the clinical utility of the BART in differentiating between bvFTD and AD patients. Using multiple BART outcome variables––including number of balloons popped, mean reaction time, total money collected, and mean adjusted pumps per trial––we hypothesised that BART performance would be impaired (e.g., more balloons popped, quicker reaction time, lower total money collected, and higher mean adjusted pumps) in both patient groups relative to controls, with worse performance in bvFTD compared with AD patients.

Second, we explored relationships between BART performance and clinical symptoms of disinhibition and apathy. We hypothesised that BART outcome variables would be correlated with informant-reported symptoms of disinhibition and apathy across patient groups. In addition, we used voxel-based morphometry to identify the neural correlates of BART performance. In line with the abovementioned studies, we expected that BART performance would be associated with the severity of OFC atrophy, irrespective of clinical diagnosis.

Finally, taking into account the heterogeneity of disinhibition and apathy symptoms in bvFTD and AD, we took a transdiagnostic approach to explore whether clinical subgroups of patients could be identified based on patterns of BART performance. We hypothesised that these subgroups of patients would show distinct profiles of disinhibition and apathy symptoms, as well as divergent patterns of brain atrophy.

## Methods

### Participants

Forty-one bvFTD patients and 28 AD patients were recruited from the FRONTIER Younger Onset Dementia Research Clinic in Sydney. All patients met clinical diagnostic criteria for probable bvFTD (Rascovsky et al., 2011) or probable AD (McKhann et al., 2011). Disease duration was estimated as the number of years since the onset of symptoms. The Clinical Dementia Rating Scale – Frontotemporal Lobar Degeneration (CDR-FTLD) (Mioshi et al., 2016) was used to assess disease severity. The Addenbrooke’s Cognitive Examination, 3rd edition (ACE-III), was used to assess general cognitive functioning (Hsieh et al., 2013).

Forty-two healthy controls were recruited from the FRONTIER research volunteer database. All control participants scored above the dementia cutoff (> 88/100) on the ACE-III (Hsieh et al., 2013).

Exclusion criteria for patients and controls included current or prior history of significant, untreated mental illness, significant head injury, movement disorders, cerebrovascular disease (stroke, transient ischaemic attacks), alcohol or other drug abuse, and limited English proficiency.

The South-Eastern Sydney Local Health District and the University of Sydney ethics committees approved the study. All participants or their person responsible provided written informed consent and the study was conducted in accordance with the Declaration of Helsinki.

### Procedure

All participants were assessed by a multidisciplinary team, consisting of a neurologist, neuropsychologists, and trained research assistants, over 1 to 2 consecutive days. Participants underwent a comprehensive clinical assessment, including neurological examination, neuropsychological assessment (including the BART, see below) and structural magnetic resonance imaging (MRI) brain scan. Patients were accompanied by their carer/informant, who completed informant reported measures (including disinhibition and apathy measures, see below). For control participants, informant-reported measures were mailed out and completed prior to their appointments.

### Measures

#### Balloon analogue risk task

All participants completed the BART on an iPad using the Joggle research platform (https://joggleresearch.com). The task began with written instructions and a practice trial. Participants who failed to understand the instructions did not continue with the task and were excluded from the study. As shown in Fig. 1, participants were asked to tap a button that pumped the balloon; each pump increased their monetary reward by $1. Participants could choose to keep pumping or tap the “collect” button, which ended the trial and added the money acquired to a permanent “bank.” If the balloon popped before they tapped “collect,” the trial ended, and they lost all the money they had accumulated for that balloon (Fig. 1). The point at which each balloon burst differed for each trial but was consistent across all participants. All participants completed 30 trials, with an average task completion time of 5 minutes. Participants did not receive any real monetary reward upon completion of the task but were not explicitly made aware of this during the task instructions.

BART performance variables of interest included 1) the total number of balloons popped (maximum = 30); 2) the mean adjusted number of pumps (in line with procedures reported in the original BART study (Lejuez et al., 2002), we excluded trials with popped balloons, because the number of pumps on these balloons was constrained and therefore limited variability across participants); 3) the total amount of money collected across the 30 trials; and 4) the average reaction time per pump response (in milliseconds) (Fig. 1).

#### Assessment of disinhibition

The Cambridge Behavioural Inventory-Revised (CBI-R) (Wear et al., 2008) abnormal behaviour subscale (6 items) was used to assess symptoms of disinhibition (e.g., “acts impulsively without thinking”). On each item, informants were asked to rate the frequency of the behaviour (from 0 = “never” to 4 = “constantly”). The maximum score is 24; higher scores indicate more severe symptoms of disinhibition. Scores were converted into percentages of the maximum score.

#### Assessment of apathy

The Dimensional Apathy Scale (DAS) (total 24 items) (Radakovic et al., 2016) was completed by informants and used to assess symptoms of apathy across executive, emotional and initiation dimensions (e.g., “they need encouragement to get things started”). Each item was rated between 0 (“hardly ever”) and 3 (“almost always/always) and summed to yield a total apathy score (maximum score = 72); higher scores indicated higher levels of apathy.

### Statistical analyses

Data were analysed by using IBM SPSS v. 28 (SPSS Inc., Chicago, IL). Shapiro–Wilk tests were first used to determine whether demographic variables, background clinical variables, and measures of interest were normally distributed. Normally distributed variables (age, DAS, BART mean adjusted pumps) were compared across groups by using ANOVAs followed by Sidak post hoc tests. Data that were not normally distributed were analysed by using Kruskal–Wallis tests followed by post hoc pairwise comparisons, using Dunn's (1964) procedure with Bonferroni correction for multiple comparisons (education, CDR-FTLD, ACE-III, BART balloons popped, mean reaction time, and total money collected) or Mann–Whitney *U* tests (disease duration). Chi-squared tests were used to compare sex distribution across groups.

To investigate differences in BART performance across controls and bvFTD and AD patients, group comparisons (ANOVAs or Kruskal–Wallis tests) with post hoc pairwise comparisons were conducted.

Taking a transdiagnostic approach, Pearson (for normally distributed variables) or Spearman (for non-normally distributed variables) correlation analyses were used to examine the relationships between performance on the BART outcome variables and measures of disinhibition (CBI-R abnormal behaviour subscale score) and apathy (DAS total score), across both dementia groups. To correct for multiple comparisons, *p* values < 0.0125 were interpreted as statistically significant.

Finally, a hierarchical cluster analysis was conducted to identify patient subgroups based on their BART performance. A hierarchical cluster analysis was chosen, because this did not require a prespecified number of groups and is more appropriate for smaller sample sizes (Ellis, 2022). The four BART outcome variables––balloons popped, mean reaction time, total money collected, and mean adjusted pumps––were entered. Individual cases were selected for groups by comparing the squared Euclidian distance and standardised by using z-scores. Group comparisons were then conducted (using ANOVAs or Kruskal–Wallis tests) to compare the groups on BART performance and measures of disinhibition (CBI-R abnormal behaviour subscale score) and apathy (DAS total score).

### Neuroimaging analysis

A total of 100 whole-brain structural MRI scans (37 bvFTD, 25 AD, 38 healthy controls) were available for the voxel-based morphometry analysis. Eleven participants (4 bvFTD; 3 AD; 4 control) were excluded due to ineligibility for MRI scanning or excessive movement artifacts. Scans were collected using a 3 T GE scanner with the following protocol: coronal orientation, matrix 256 × 256, 200 slices, 1-mm^2^ in-plane resolution, 1-mm slice thickness, echo time/repetition time: 2.5/6.7 ms, flip angle 8°.

Magnetic resonance imaging data were analysed using FSL-VBM, a VBM analysis, which is part of the FMRI software package (Ashburner & Friston, 2000). Following brain extraction, tissue segmentation was performed by using FMRIB’s Automatic Segmentation Tool (Zhang et al., 2001). The resulting grey matter partial volume maps were aligned to the Montreal Neurological Institute standard space (MNI52) by using the nonlinear registration approach with FNIRT (Andersson et al., 2007a, 2007b), which uses a b-spline representation of the registration warp field (Rueckert et al., 1999). A study-specific template was created to which the native grey matter images were nonlinearly re-registered. Modulation of the registered partial volume maps were performed by dividing them by the Jacobian of the warp field. The modulated, segmented images were smoothed with an isotropic Gaussian kernel with a sigma of 3 mm.

Voxel-wise general linear models (GLM) were used to investigate differences in grey matter intensity via permutation-based nonparametric testing (Nichols & Holmes, 2002) with 5,000 permutations per contrast. Group differences in grey matter intensity were tested for significance at *p* < 0.05, corrected for multiple comparisons via Family-Wise Error correction across space. A cluster extent threshold of 200 contiguous voxels was applied for group comparisons. Next, separate whole-brain covariate analyses were conducted to explore the relationships between grey matter intensity and the four BART performance variables (balloons popped, mean adjusted pumps, total money collected and mean reaction time). Covariate analyses were family-wise error corrected at *p* < 0.05, with a cluster extent threshold of 200 contiguous voxels. Finally, differences in grey matter intensity between controls and groups A-D, identified through the hierarchical cluster analysis, were tested for significance at *p* < 0.05, corrected for multiple comparisons via Family-Wise Error correction across space, with a cluster extent threshold of 200 contiguous voxels. Anatomical locations of significant results were overlaid on the MNI standard brain, with maximum coordinates provided in MNI stereotaxic space. Anatomical labels were determined with reference to the Harvard Oxford probabilistic cortical and subcortical atlases.

## Results

### Demographic and background clinical measures

Demographic and background clinical variables for each group are reported in Table 1. A significant group difference in age was observed *H*(2) = 4.12, *p* = 0.02, such that bvFTD patients were significantly younger than controls, *p* = 0.02. However, there was no significant age difference between controls and AD patients, *p* = 0.74, or bvFTD and AD patients, *p* = 0.28 (Table 1). The groups did not differ in terms of sex distribution, *χ*^*2*^(2) = 3.08, *p* = 0.215. A significant group difference in years of education was also evident, *H*(2) = 9.18, *p* = 0.01, such that controls had completed significantly more years of education than bvFTD patients, *p* = 0.003, while controls and AD patients, *p* = 0.32, and AD and bvFTD patients, *p* = 0.09, completed similar years of education.

**Table 1:** Demographic, background clinical, disinhibition, and apathy variables across the control, bvFTD, and AD groups

	Mean (SD)			Overall group differences	Post hoc group comparisons
	Controls	bvFTD	AD		
N	42	41	28		
Sex (M/F)	22/20	29/12	16/12	*χ*^*2*^(2) = 3.08, *p* = 0.215	-
Age (years)	66.75 (5.67)	62.24 (8.37)	65.14 (7.65)	*H*(2) = 4.12, *p* = 0.02	C > bvFTD
Education (years)	14.46 (3.04)	12.79 (2.95)	13.66 (2.79)	*H*(2) = 9.18, *p* = 0.01	C < bvFTD
Disease duration (years)	-	5.05 (3.19)	4.39 (2.27)	*H*(3) = 5.04, *p* = 0.169	-
CDR-FTLD [24]	0.32 (0.52)	7.59 (3.87)	5.96 (2.26)	*H*(2) = 77.13, *p* < 0.001	C < AD, bvFTD
ACE-III [100]	94.81 (3.64)	76.47 (14.18)	63.79 (16.62)	*H*(2) = 67.99, *p* < 0.001	C > bvFTD > AD
CBI-R abnormal behaviour [100]	3.96 (4.93)	41.45 (20.89)	16.52 (13.25)	*H*(2) = 62.47, *p* < 0.001	C < AD < bvFTD
DAS [72]	17.89 (9.76)	51.16 (8.94)	43.26 (10.80)	*F*(2) = 118.72, *p* < 0.001	C < AD < bvFTD

SD = standard deviation; C = controls; ACE-III = Addenbrooke’s Cognitive Examination, 3rd edition; CDR-FTLD = Clinical Dementia Rating Scale – Frontal Lobar Degeneration; CBI-R = Cambridge Behavioural Inventory Revised; DAS = Dimensional Apathy Scale; Values in square brackets indicate maximum scores. Statistically significant post hoc group differences summarised in the far-right column

A significant group difference was observed on the cognitive screening test (ACE-III), *H*(2) = 67.99, *p* < 0.001, with controls outperforming both the bvFTD, *p* = 0.03, and AD, *p* < 0.001, groups, and bvFTD patients outperforming AD patients, *p* = 0.03. Regarding disease severity, significant group differences were evident on the CDR-FTLD, *H*(2) = 77.13, *p* < 0.001, with controls showing significantly lower scores than both bvFTD, *p* < 0.001, and AD patients, *p* < 0.001, and bvFTD and AD patients showing similar levels of impairment, *p* = 0.27. Similarly, bvFTD and AD patients did not show significant differences in disease duration, *H*(3) = 5.04, *p* = 0.169.

### Assessment of disinhibition and apathy

A significant group difference was observed on the CBI-R abnormal behaviour subscale *H*(2) = 62.47, *p* < 0.001, with bvFTD patients, *p* < 0.001, and AD patients, *p* < 0.001, presenting with more symptoms of disinhibition relative to controls, and bvFTD patients showing more disinhibition than AD patients, *p* < 0.001.

A significant difference was found between groups on the DAS, *F*(2) = 118.72, *p* < 0.001, with greater apathy in bvFTD patients, *p* < 0.001, and AD patients, *p* < 0.001, relative to controls and greater apathy in bvFTD compared with AD patients, *p* = 0.01.

### BART performance across groups

**Fig. 2:**
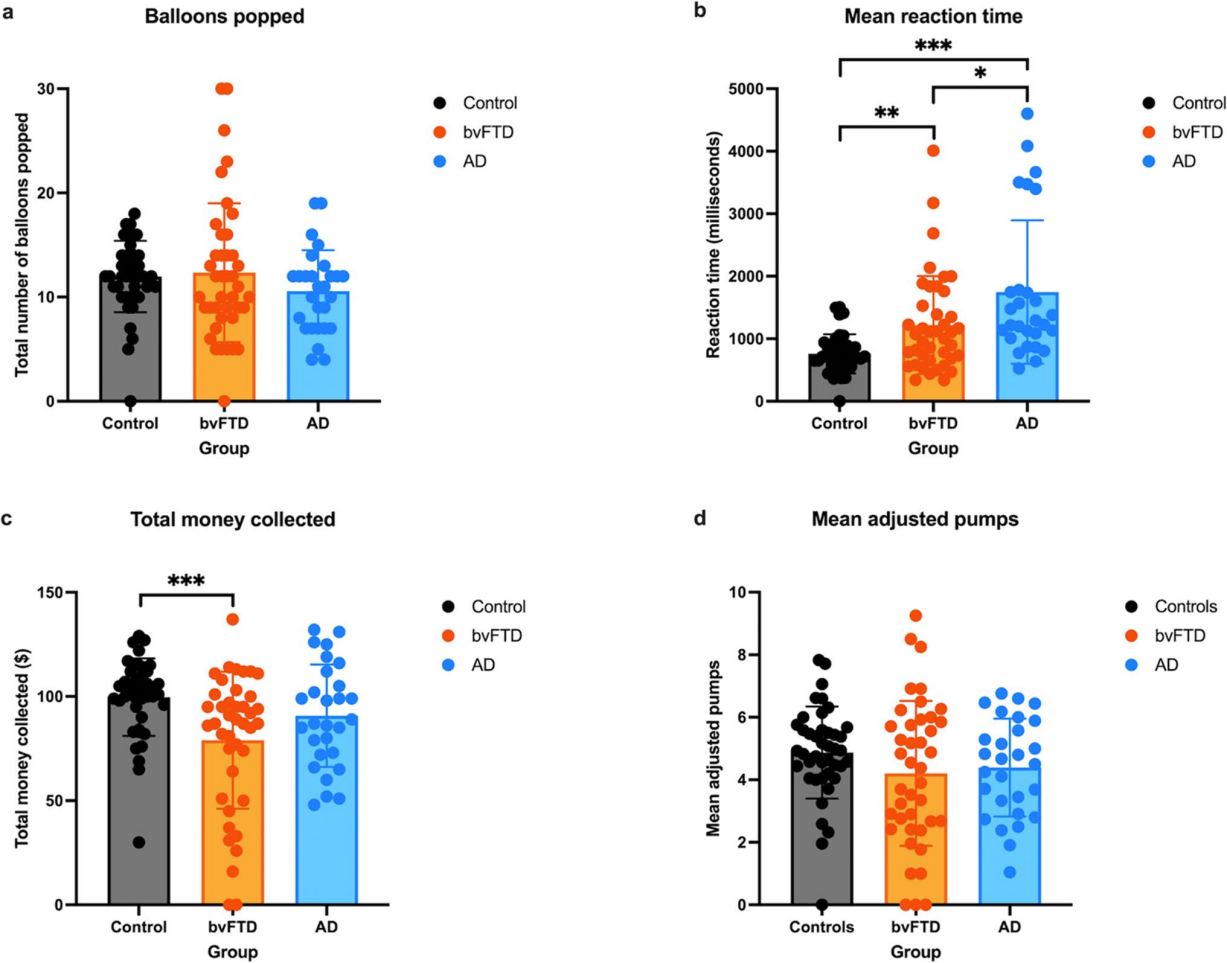
Means and individual scores across diagnosis group for BART variables. a. Balloons popped. **b.** Mean reaction time. **c.** Total money collected. **d.** Mean adjusted pumps. *p < 0.05; **p < 0.01; ***p < 0.001

One-way ANOVAs or Kruskal–Wallis tests were used to compare the four main BART variables across groups (Fig. 2). A statistically significant difference between all three groups was found for mean reaction time, *H*(2) = 25.92, *p* < 0.001, with AD patients responding slower than both controls, *p* < 0.001, and bvFTD patients, *p* = 0.03, and bvFTD patients responding slower than controls, *p* = 0.002 (Fig. 2b). While there was an overall group difference in total money collected, *H*(2) = 11.25, *p* = 0.004, this appeared to be driven by bvFTD patients collecting significantly less money than controls, *p* < 0.001 (Fig. 2c). The total money collected did not significantly differ between controls and AD, *p* = 0.07, or between AD and bvFTD patients, *p* = 0.25. In contrast, no significant group differences were observed on the total number of balloons popped, *H*(2) = 3.22, *p* = 0.199 (Fig. 2a), or mean adjusted pumps, *F*(2) = 1.41, *p* = 0.248 (Fig. 2d). In summary, bvFTD patients only performed worse than controls in terms of total money collected, whereas AD patients showed significantly slower mean reaction times relative to the other two groups.

Given that the AD patients performed significantly lower than bvFTD patients on the cognitive screening test (ACE-III), group comparisons were re-run using ANCOVAs, with ACE-III scores included as a covariate. When controlling for cognitive impairment, none of the group differences in total balloons popped, *F*(2) = 0.76, *p* = 0.47, mean reaction time, *F*(2) = 1.55, *p* = 0.22, or mean adjusted pumps, *F*(2) = 0.59, *p* = 0.56, were statistically significant. However, the group difference in total money collected remained statistically significant, *F*(2) = 4.23, *p* = 0.02, although post hoc pairwise comparisons indicated that bvFTD patients collected significantly less money than AD patients, *p* = 0.02, but a similar amount to controls, *p* = 0.66.

As shown in Fig. 2, BART scores were highly variable. In particular, two patients with bvFTD popped all 30 balloons, while one patient with bvFTD popped no balloons and collected $0 (Figs. 2a and c). Furthermore, two bvFTD participants and six AD participants had mean reaction times > 3000 ms (Fig. 2b). Because these response patterns may reflect clinically meaningful BART performance, these participants were not excluded from the analyses.

### Relationships between BART performance and symptoms of apathy and disinhibition

Taking a transdiagnostic approach, we conducted correlational analyses across all patients (n = 69), excluding controls, to examine the relationships between BART performance and symptoms of apathy and disinhibition, regardless of dementia diagnosis. As shown in Table 2, disinhibition was positively correlated with total balloons popped. Disinhibition was also negatively correlated with mean reaction time. Similarly, apathy was positively correlated with total balloons popped but negatively correlated with mean reaction time.

**Table 2:** Correlations between BART variables and measures of apathy and disinhibition in bvFTD and AD patients

BART performance variables	Disinhibition (CBI-R abnormal behaviour subscale)	Apathy (DAS)
Total balloons popped	**r**_**S**_**(64) = 0.330, *p* = 0.007**	**r**_**S**_**(63) = 0.410, *p* < 0.001**
Mean reaction time	**r**_**S**_**(64) = − 0.324, *p* = 0.008**	**r**_**S**_**(63) = − 0.379, *p* = 0.002**
Total money collected	r_S_(64) = 0.007, *p* = 0.956	r_S_(63) = − 0.166, *p* = 0.185
Mean adjusted pumps	r_S_(64) = 0.257, *p* = 0.037	r_P_(63) = 0.120, *p* = 0.339

Statistically significant correlations in bold. Correlations are significant at *p* < 0.0125

*r*_*P*_   Pearson’s correlation, *r*_*S*_ Spearman’s correlation, *CBI-R *Cambridge Behavioural Inventory – Revised, *DAS* Dimensional Apathy Scale

Degrees of freedom in brackets.

Partial correlation analyses were used to further explore whether overall level of cognitive impairment (ACE-III total score) or dementia severity (CDR-FTLD) could have explained the significant correlations between BART performance variables and disinhibition or apathy. Mean reaction time still correlated significantly with disinhibition, r(64) =  − 0.398, *p* = 0.002, and apathy, r(63) =  − 0.451, *p* < 0.001, when overall cognitive impairment was considered. The total number of balloons popped also remained significantly associated with apathy, r(63) = 0.454, *p* < 0.001, and showed a trend towards a significant correlation with disinhibition, r(64) = 0.307, *p* = 0.021, after controlling for overall cognitive impairment (Supplementary Table 1). Similarly, when accounting for dementia severity, the total number of balloons popped and mean reaction time remained significantly associated with both disinhibition (total balloons popped: r(64) = 0.319, *p* = 0.012; mean reaction time: r(64) =  − 0.407, *p* = 0.001) and apathy (total balloons popped: r(64) =  − 0.407, *p* = 0.001; mean reaction time: r(63) =  − 0.462, *p* < 0.001) (Supplementary Table 2).

### Hierarchical cluster analysis

**Fig. 3:**
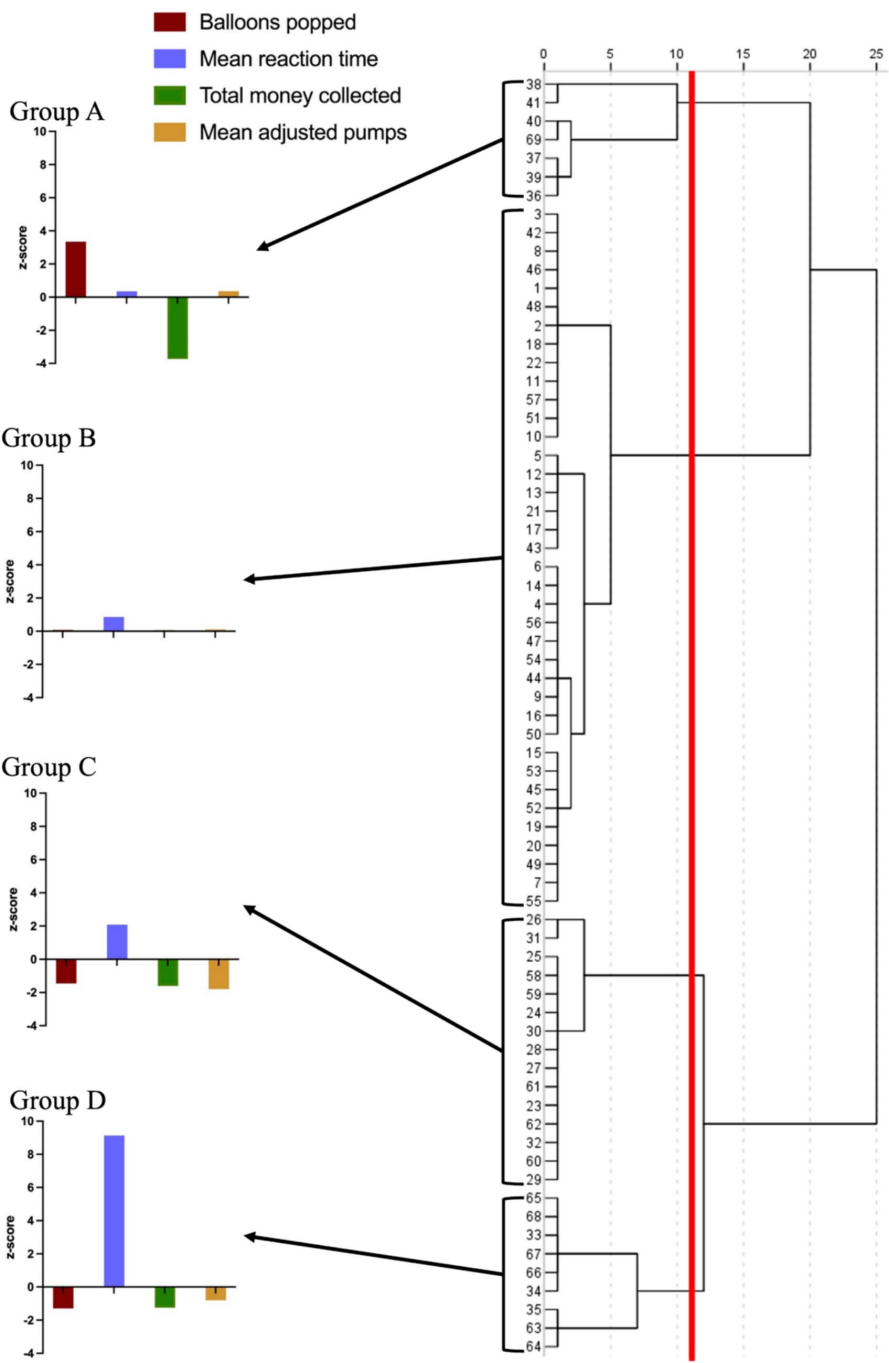
Dendrogram using Ward Linkage depicting the arrangement obtained by a hierarchical cluster analysis of bvFTD and AD patients. The x-axis represents the clustering distance. The vertical red line indicates the cutpoint that divides the sample into 4 clusters. BART variables were converted to z-scores for illustrative purposes (see bar graphs on the left)

A hierarchical cluster analysis was performed across all bvFTD and AD patients, using Ward’s method, by inputting the main BART variables—balloons popped, mean reaction time, total money collected, and mean adjusted pumps—into the analysis. Individual cases were sorted into groups by comparing the squared Euclidian distance and were standardised using z-scores. The cutoff point for the number of clusters was determined by visual inspection of the dendrogram and agglomeration schedule, resulting in four distinct groups (Fig. 3).

#### Comparison of BART variables and measures of disinhibition and apathy across clinical subgroups

**Fig. 4:**
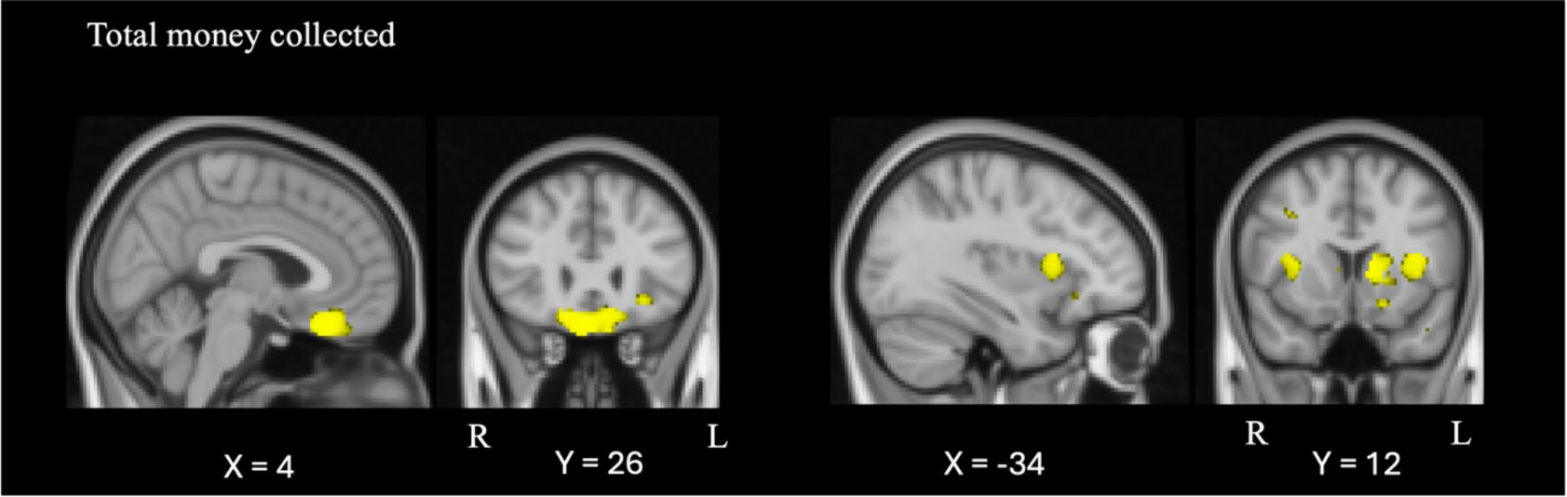
Regions of grey matter atrophy correlating with BART total money collected. Coloured voxels show regions that were statistically significant at *p* < 0.05, family-wise error corrected, with a cluster threshold of 200 contiguous voxels. Clusters are overlaid on the MNI standard brain

The four groups identified through the hierarchical cluster analysis did not differ in age, gender distribution, education, disease duration, or disease severity (CDR-FTLD) *p* ≥ 0.08 (Supplementary Table 3). However, group D had significantly lower ACE-III scores than group C, *p* = 0.04, with no significant differences between the other groups, *p* ≥ 0.06. As expected, comparison of BART variables across controls and the four groups identified through the hierarchical cluster analysis yielded statistically significant differences (Table 3; Fig. 4). The groups also showed significant differences on measures of disinhibition and apathy (Table 3).

**Table 3:**
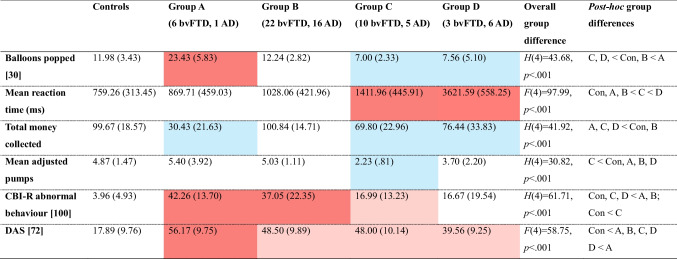
BART outcome variables and symptoms of disinhibition and apathy in controls and patient groups identified through the hierarchical cluster analysis

Scores represent means with standard deviations in brackets; Values in square brackets indicate maximum scores; CBI-R: Cambridge Behavioural Inventory-Revised, DAS: Dimensional Apathy Scale. *Post hoc* group differences summarised in the far-right column (Con = controls) significant at *p*<.05. Colours represent significant *post hoc* group comparisons (red = significantly higher than controls and other patient groups; pink = significantly higher scores than controls; white = no significant difference from controls; blue = significantly lower scores than controls and other patient groups)

#### Group A

Group A, comprised of six patients with bvFTD and only one patient with AD, showed the greatest impairment on the BART, including the highest number of balloons popped, compared with all other groups, *p* < 0.001, and lowest total money collected, relative to all other groups, *p* ≤ 0.05, except for group C, *p* = 0.18.

Group A patients showed the most severe disinhibition symptoms (CBI-R abnormal behaviour score), which were significantly higher than groups C and D, *p* ≤ 0.03. Group A also presented with the most elevated apathy symptoms (DAS), although this was only significantly higher relative to group D, *p* = 0.02 (see Supplementary Materials for all detailed pairwise comparisons).

#### Group B

Group B, comprised of 22 bvFTD and 16 AD patients, performed closest to controls, collecting a similar amount of money, which was significantly more than the other three patient groups, *p* ≤ 0.02. Patients in group B were also similar to controls in terms of the number of balloons popped, *p* = 0.87, and mean adjusted pumps, *p* = 0.66, although their mean reaction time was slower than controls, *p* = 0.04.

Despite performing similarly to controls on the BART, group B patients presented with symptoms of disinhibition that were significantly higher than controls, *p* < 0.001, and two of the three other patient groups (versus group C, *p* = 0.02, group D, *p* = 0.01, group A, *p* = 0.52). Furthermore, group B patients showed greater apathy than controls, *p* < 0.001, although this did not differ relative to the other patient groups, *p* ≥ 0.15.

#### Group C

Group C, which comprised of ten patients with bvFTD and five patients with AD, popped significantly fewer balloons than groups B and A, *p* < 0.001, and showed reaction times that were significantly faster than group D, *p* < 0.001, but slower than groups A and B, *p* ≤ 0.04. This group also collected significantly less money than group B, *p* < 0.001, and had the lowest mean adjusted pumps, which was significantly lower than groups B and A, *p* < 0.001.

Group C patients showed higher disinhibition (CBI-R abnormal behaviour) than controls,* p* = 0.01, but lower disinhibition than groups B, *p* = 0.02, and A, *p* = 0.03, but not D, *p* = 0.60. Finally, they did not significantly differ from any other patient groups on the DAS, *p* ≥ 0.42, but had higher apathy than controls, *p* < 0.001.

#### Group D

Group D, which comprised of three patients with bvFTD and six patients with AD, showed largely similar BART performance to group C, with the exception of mean reaction time, which was significantly slower than all other groups, *p* < 0.001.

On measures of disinhibition, group D patients showed similar levels of disinhibition (CBI-R abnormal behaviour score) to controls, *p* = 0.07, and group C, *p* = 0.06, and significantly lower disinhibition than groups B, *p* = 0.01, and A, *p* = 0.01. On measures of apathy, group D showed higher DAS scores compared with controls, *p* < 0.001, but these symptoms were similar to group B,* p* = 0.18, and group C, *p* = 0.42, and lower than group A,* p* = 0.02.

### Neuroimaging results

#### Diagnosis group differences in grey matter atrophy

The control, bvFTD, and AD groups were contrasted to identify diagnosis group-specific patterns of atrophy. Overall, patterns of atrophy were in line with those previously reported for AD (McKhann et al., 2011), with patients showing greater atrophy in the bilateral medial temporal lobe, extending to the medial parietal and temporooccipital cortices bilaterally. BvFTD patients also showed characteristic patterns of atrophy (Rosen et al., 2002), encompassing the medial frontal and orbitofrontal cortices and basal ganglia, and extending towards the medial temporal and medial parietal lobes bilaterally (Supplementary Table 4; Figs. 1 and 2).

#### Grey matter correlates of BART performance

Table 4 and Fig. 4 show the results from the VBM analyses exploring associations between grey matter intensity decrease and BART performance on measures of total money collected, mean reaction time, total balloons popped, and mean adjusted pumps. Combining all participants together, lower total money collected was associated with greater atrophy in the subcallosal cortex bilaterally, extending into the frontal medial cortices and orbitofrontal cortices, as well as the left frontal operculum cortex extending into the insular cortex (Fig. 4). No other BART variables were associated with significant grey matter intensity reduction.

**Table 4:** Voxel-based morphometry results showing regions of significant grey matter atrophy that covary with BART performance on measures of total money collected, mean reaction time, total balloons popped and mean adjusted pumps

		MNI coordinates			
Regions	Hemisphere	X	Y	Z	Cluster size (number of voxels)
**Total money collected**					
Subcallosal cortex; frontal medial cortex; orbitofrontal cortex	Bilateral	4	26	− 22	1856
Frontal operculum cortex; insular cortex	Left	− 34	12	12	212
**Mean reaction time**					
-					
**Total balloons popped**					
-					
**Mean adjusted pumps**					
-					

All results FWE-corrected at *p* < 0.05; only clusters with at least 200 contiguous voxels reported. MNI = Montreal Neurological Institute.

### Patterns of atrophy across groups

Next, patient groups identified through the hierarchical cluster analysis were contrasted to identify patterns of atrophy.

#### Group A

**Fig. 5:**
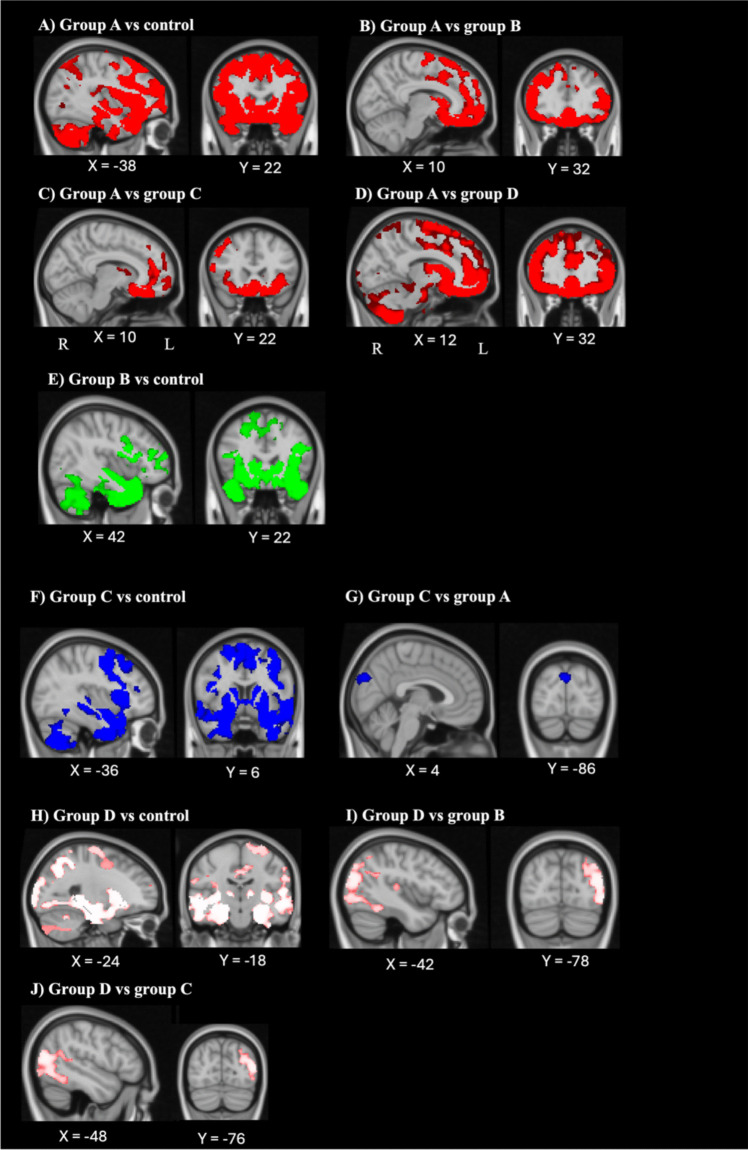
Regions of significant grey matter atrophy in **A**) group A vs. controls, **B**) group A vs. group B, **C**) group A vs. group C, **D**) group A vs. group C, **E**) group B vs. control, **F**) group C vs. control, **G**) group C vs. group A, **H**) group D vs. control, **I**) group D vs. group B and **J**) group D vs. group C. Coloured voxels show regions that were statistically significant at *p* < 0.05, family-wise error corrected, with a cluster threshold of 200 contiguous voxels. Clusters are overlaid on the MNI standard brain

Compared with controls, group A showed significant atrophy in the left temporal pole, extending into the inferior temporal gyrus, the parahippocampal gyrus, the temporal fusiform cortex, insular cortex, planum polare, frontal pole, and orbitofrontal cortex (Table 5; Fig. 5A).

**Table 5:** Voxel-based morphometry results showing regions of significant grey matter atrophy for group A patients relative to other patient groups and controls

		MNI coordinates			
Regions	Hemisphere	X	Y	Z	Cluster size (number of voxels)
**Group A vs. controls**					
Temporal pole; inferior temporal gyrus; parahippocampal gyrus; temporal fusiform cortex; insular cortex; planum polare; frontal pole; orbitofrontal cortex	Left	− 38	22	40	87,971
**Group A vs. group B**					
Frontal pole; orbitofrontal cortex; frontal medial cortex; subcallosal cortex; anterior cingulate gyrus; paracingulate gyrus	Bilateral	10	32	− 28	31,214
Superior lateral occipital cortex; angular gyrus; posterior supramarginal gyrus	Left	− 48	− 64	38	749
Anterior middle temporal gyrus; anterior superior temporal gyrus; temporal pole	Right	46	0	− 26	296
Posterior inferior temporal gyrus; temporooccipital inferior temporal gyrus; posterior middle temporal gyrus; parahippocampal gyrus	Right	62	− 40	− 26	292
**Group A vs. group C**					
Orbitofrontal cortex; frontal pole; frontal medial cortex; frontal operculum cortex; insular cortex; subcallosal cortex; anterior cingulate gyrus; paracingulate gyrus	Bilateral	10	22	− 26	15,129
**Group A vs. group D**					
Orbitofrontal cortex; frontal pole; frontal gyrus; pars triangularis; frontal medial cortex; paracingulate gyrus; subcallosal cortex; anterior cingulate gyrus	Bilateral	12	32	− 28	17,851

All results FWE-corrected at *p* < 0.05; only clusters with at least 200 contiguous voxels reported. MNI = Montreal Neurological Institute.

Group A also showed extensive frontal and temporal atrophy compared with the other three patient groups. Relative to group B, group A showed greater atrophy in the bilateral frontal pole, extending to the orbitofrontal cortices, frontal medial cortices, subcallosal cortices, anterior cingulate gyri, and paracingulate gyri. There was also greater atrophy in the left superior lateral occipital cortex, angular gyrus, and posterior supramarginal gyrus, as well as the right anterior middle temporal gyrus, extending to the anterior superior temporal gyrus, and the temporal pole. The right posterior inferior temporal gyrus also showed greater atrophy, extending into the temporooccipital inferior temporal gyrus, posterior middle temporal gyrus, and parahippocampal gyrus (Table 5; Fig. 5B).

Compared with group C, group A showed greater atrophy in the bilateral orbitofrontal cortices, extending into the frontal pole, the frontal medial cortices, the frontal operculum cortices, insular cortices, subcallosal cortices, anterior cingulate gyri, and paracingulate gyri (Table 5; Fig. 5C). Finally, compared with group D, group A showed greater atrophy in the bilateral orbitofrontal cortices, extending into the frontal pole, the frontal gyri, pars triangularis, the frontal medial cortices, the paracingulate gyri, subcallosal cortices, and the anterior cingulate gyri (Table 5; Fig. 5D).

#### Group B

As shown in Table 6 and Fig. 5E, patients in group B showed greater atrophy than controls in the right temporal pole, extending to the posterior superior temporal gyrus, the right middle and inferior temporal gyri, the temporal fusiform cortex, anterior parahippocampal gyrus, and planum polare, as well as the frontal pole and orbitofrontal cortices. Regions of greater atrophy were also identified in the right precuneus cortex, extending into the superior lateral occipital cortex, the postcentral gyrus, and the superior parietal lobe. Relative to groups A, C, and D, those in group B did not show any regions of significantly greater atrophy.

**Table 6:** Voxel-based morphometry results showing regions of significant grey matter atrophy for group B patients relative to other patient groups and controls

		MNI coordinates			
Regions	Hemisphere	X	Y	Z	Cluster size (number of voxels)
**Group B vs. controls**					
Temporal pole; posterior superior temporal gyrus; middle and inferior temporal gyrus; temporal fusiform cortex; anterior parahippocampal gyrus; planum polare; frontal pole; orbitofrontal cortex	Right	42	22	− 36	46,322
Precuneus cortex; superior lateral occipital cortex; postcentral gyrus; superior parietal lobe	Right	10	− 48	58	220
**Group B vs. group A**					
-					
**Group B vs. group C**					
-					
**Group B vs. group D**					
-					

All results FWE-corrected at *p* < 0.05; only clusters with at least 200 contiguous voxels reported. MNI = Montreal Neurological Institute.

#### Group C

Relative to controls, group C showed greater atrophy in the left temporal pole, extending to the anterior inferior temporal gyrus, the parahippocampal gyrus, the temporal fusiform cortex, the lingual gyrus, the insular cortex, the subcallosal cortex, frontal medial cortex, the frontal pole and the orbitofrontal cortex (Table 7; Fig. 5F). Group C also showed greater atrophy than group A in the bilateral supracalcarine cortices, intracalcarine cortices, cuneal cortices, precuneal cortices, extending into the occipital fusiform gyrus and occipital pole (Table 7; Fig. 5G).

**Table 7:** Voxel-based morphometry results showing regions of significant grey matter atrophy for group C patients relative to other patient groups and controls

		MNI coordinates			
Regions	Hemisphere	X	Y	Z	Cluster size (number of voxels)
**Group C vs. controls**					
Temporal pole; anterior inferior temporal gyrus; parahippocampal gyrus; temporal fusiform cortex; lingual gyrus; insular cortex; subcallosal cortex; frontal medial cortex; frontal pole; orbitofrontal cortex	Left	− 36	6	− 42	49,198
**Group C vs. group A**					
Supracalcarine cortex; intracalcarine cortex; cuneal cortex; precuneal cortex; occipital fusiform gyrus; occipital pole	Bilateral	4	− 86	30	319
**Group C vs. group B**					
-					
**Group C vs. group D**					
-					

All results FWE-corrected at *p* < 0.05; only clusters with at least 200 contiguous voxels reported. MNI = Montreal Neurological Institute.

#### Group D

Compared with controls, group D showed greater atrophy in the left anterior parahippocampal gyrus, anterior middle, and inferior temporal gyri, extending into the temporal pole, the fusiform cortex (posterior temporal, temporal occipital, and occipital), as well as the lingual gyrus, insular cortex, inferior frontal gyrus, and orbitofrontal cortex (Table 8; Fig. 5H). Comparisons between the patient groups indicated greater atrophy in group D compared with group B in the left superior lateral occipital cortex, extending to the inferior lateral occipital cortex, the angular gyrus, the temporal occipital fusiform cortex, and temporooccipital and posterior inferior temporal gyrus, the angular gyrus and posterior supramarginal gyrus (Table 8; Fig. 5I). Furthermore, group D also showed greater atrophy than group C in the left inferior lateral occipital cortex, extending into the superior lateral occipital cortex, the angular gyrus, posterior supramarginal gyrus, and the temporooccipital middle and inferior temporal gyri (Table 8; Fig. 5J).

**Table 8:** Voxel-based morphometry results showing regions of significant grey matter atrophy for group D patients relative to other patient groups and controls

		MNI coordinates			
Regions	Hemisphere	X	Y	Z	Cluster size (number of voxels)
**Group D vs. controls**					
Anterior parahippocampal gyrus; anterior middle temporal gyrus; anterior inferior temporal gyrus; temporal pole; posterior temporal fusiform cortex; temporal occipital fusiform cortex; occipital fusiform gyrus; lingual gyrus; insular cortex; inferior frontal gyrus; orbitofrontal cortex	Left	− 24	− 18	− 32	52,356
**Group D vs. group A**					
**-**					
**Group D vs. group B**					
Superior lateral occipital cortex; lateral occipital cortex; angular gyrus; temporal occipital fusiform cortex; temporooccipital and posterior inferior temporal gyrus; angular gyrus; posterior supramarginal gyrus	Left	− 42	− 78	18	7308
**Group D vs. group C**					
Inferior lateral occipital cortex; lateral occipital cortex; angular gyrus; posterior supramarginal gyrus; temporooccipital middle and inferior temporal gyri	Left	− 48	− 76	10	2319

All results FWE-corrected at *p* < 0.05; only clusters with at least 200 contiguous voxels reported. MNI = Montreal Neurological Institute.

## Discussion

This study examined affective decision-making using the BART in bvFTD and AD patients, and a healthy control group. Importantly, we included multiple BART outcome variables in our analyses, which allowed more nuanced insights into each group’s performance profile, associations with symptoms of disinhibition and apathy, as well as the neural correlates of BART performance. We consider the theoretical and clinical implications of these findings in more detail below.

### BART performance in bvFTD and AD

When comparing performance between patient groups and controls, some key differences were observed in total money collected and mean reaction time, though these differences were less pronounced than expected. In particular, bvFTD patients collected the least amount of money on the BART. This is largely consistent with results from the Strenziok et al. (2011) BART study, which found that bvFTD patients showed consistently lower mean adjusted pumps across all 30 trials and by extension, lower total winnings. In contrast, controls gradually learned to pump the balloons more, thus increasing their mean adjusted pumps and total winnings (Strenziok et al., 2011). While we did not find significant differences in mean adjusted pumps in our study, this could potentially be explained by the lower average burst point in our version of the BART, which limited the range in responses, compared to the version of the task used in the Strenziok et al. (2011) study. Nonetheless, while bvFTD patients across both the current and previous study collected significantly less money, this could have been owing to either popping a large number of balloons (and thus losing the accumulated monetary reward) or pumping each balloon less (and thus “collecting” the smaller reward). Of interest, our group comparisons of the total balloons popped and mean adjusted pumps variables did not provide strong evidence in support of either of these maladaptive decision-making styles but rather indicated a high degree of variability within each patient group. We therefore explored these profiles of performance in more detail through our hierarchical cluster analysis.

Mean reaction time also emerged as a significant difference between patient groups, with AD patients showing much slower responses on the BART. While the two patient groups did not differ in terms of disease duration or severity, it is important to note that the AD group showed greater overall cognitive impairment on the ACE-III. Indeed, our results indicate that after controlling for cognitive impairment, differences in mean reaction time were no longer statistically significant. This suggests that the slower response times in AD patients may be largely explained by overall cognitive impairment, rather than deficits in affective decision-making per se.

### Relationship between BART performance and measures of disinhibition and apathy

This study also examined associations between performance on the BART and informant-reported measures of disinhibition and apathy in bvFTD and AD patients. In line with previous studies in adolescents (Hopko et al., 2006; Lejuez et al., 2002, 2003), we found that those who had more severe symptoms of disinhibition chose to inflate the balloons more, with more balloons popped and faster mean reaction times. Importantly, these correlations remained significant after controlling for overall cognitive impairment and disease severity, suggesting that these relationships did not simply reflect worsening cognitive symptoms or disease progression. As such, disinhibition was associated with a pattern of BART performance whereby the potential for higher reward outweighed the risk of popping the balloon and losing the reward. Furthermore, the relationship between greater disinhibition and faster reaction time on the BART is consistent with previous studies that have shown that informant-reported measures of disinhibition in AD and bvFTD are associated with faster reaction times on measures such as the Go/No-go task (Dubois et al., 2000; Migliaccio et al., 2020). In the context of risk-taking behaviour, links between disinhibition and disordered gambling (Manes et al., 2010; Tondo et al., 2017) and criminal risk behaviours (e.g., physical/verbal assault, financial/professional recklessness and inappropriate sexual behaviour) (Kumfor et al., 2024) have been proposed. Given that we did not include measures of risk-taking behaviours in everyday life, future studies should explore whether a disinhibited pattern of performance on the BART can serve as a useful indicator of propensity towards risk-taking behaviours.

Unexpectedly, we found that faster reaction time and more balloons popped were also associated with more severe symptoms of apathy, even after accounting for overall cognitive impairment and disease severity. Given that apathy has been associated with impaired reward learning in AD and bvFTD patients (Wong et al., 2023), it was initially predicted that apathetic patients would show more “collect” responses, resulting in fewer popped balloons. Instead, our results point to broader difficulties in affective decision-making, extending to difficulty in appropriately weighing up risks versus rewards. It is possible that apathetic patients in our study tended to respond quickly, pump more and thus, pop more balloons, due to lower sensitivity to the potential positive (winning money) and negative (popping the balloon and losing money) outcomes on the BART. Because the participants in our study did not receive actual monetary rewards, it is unclear whether apathetic patients would respond in a similar manner for larger or more tangible outcomes. Future studies are therefore needed to replicate our findings, and to explore whether outcome saliency or tangibility moderates the relationship between apathy and BART performance. Nonetheless, our findings highlight the importance of considering symptoms of both disinhibition and apathy in the context of multiple task outcome measures when investigating performance on affective decision-making tasks like the BART.

### Regions of atrophy associated with BART performance

Our neuroimaging analysis revealed a set of fronto-insular regions underpinning performance on the BART. Specifically, lower total money collected was associated with atrophy in the bilateral OFC, subcallosal and medial frontal cortices, and the left insula cortex. This is consistent with previous studies that have linked these brain regions to BART performance. In particular, Strenziok et al. (2011) found that lower change in mean adjusted pumps over trials (i.e., poorer learning of optimal decision-making strategies) on the BART correlated with lateral OFC atrophy in bvFTD patients. Similarly, an fMRI study in adolescents found that riskier behaviour on the BART (higher mean adjusted pumps) was associated with bilateral activation in the medial prefrontal and insular cortices (Chiu et al., 2012). The OFC has been widely implicated in reward-related decision-making, where it plays a critical role in encoding reward value representations that are integrated by regions such as the ventromedial prefrontal cortex and subcallosal cortex to drive choice value decision-making and action–outcome learning (Rolls, 2023). The OFC also projects to the insula, which is involved in autonomic responses that elicit emotions (Rolls, 2023). Our findings therefore indicate that degeneration of the OFC and associated regions in dementia affects the ability to learn and adapt actions in response to reward/emotion-relevant contingencies, thus impacting the affective decision-making process when weighing up potential risks versus rewards.

### Clinical subgroups of patients based on BART performance

Turning to our hierarchical cluster analysis, we found that discrete patient subgroups could be identified on the basis of patterns of BART performance. These subgroups also showed varying degrees of disinhibition and apathy symptoms and distinct atrophy profiles, although they were largely similar in terms of demographics (age, sex distribution, years of education), disease duration and disease severity (CDR-FTLD), with the exception of overall cognitive impairment, which was most severe in group D. We discuss the profiles of each group in relation to each other, and controls, in more detail below.

Group A was the only group that showed a “classically disinhibited” profile of performance on the BART. This group, primarily comprised of bvFTD patients, popped the most balloons and collected the least amount of money, although their mean reaction time and mean adjusted pumps were similar to controls. On the informant-report measures, group A patients showed the highest levels of both disinhibition and apathy. Their neuroimaging profile showed more OFC and medial frontal atrophy than any other group. As discussed earlier, these regions play key roles in reward-related decision making. These regions have also been associated with both disinhibition and apathy in previous neuroimaging studies of AD and bvFTD patients (Hornberger et al., 2011; Wei et al., 2019; Wong et al., 2023). Given their “classically disinhibited” BART performance, it seems contradictory that these patients also presented with high levels of apathy. As discussed above, it is plausible that reduced sensitivity to positive and negative outcomes in apathetic patients may also underpin the type of BART performance we would expect to see in disinhibited patients (i.e., pumping and popping more balloons, resulting in lower overall money collected). Indeed, the co-occurrence of disinhibition and apathy and their overlapping neural correlates has previously been reported in bvFTD (Lansdall et al., 2017). Furthermore, evidence from large population datasets suggests that apathy and disinhibition do not exist at either end of a continuum but rather, frequently coexist, such that individuals may have difficulty self-initiating behaviour (i.e., apathy) but when presented with simple action options, may show an increase in automatic responses to salient external stimuli (i.e., disinhibition/impulsivity) (Petitet et al., 2021). As such, the group A patients in our study may present with severe disinhibition and apathy symptoms across different everyday contexts, but when faced with simple action options, such as pressing a button to maximise rewards, tend to respond in a “classically disinhibited” manner. In terms of symptom management, understanding the circumstances under which these patients may switch between highly apathetic and highly disinhibited behaviours would be important for therapeutic approaches that rely on environmental modifications.

Group B showed virtually similar BART performance to controls, except for slightly slower reaction time. Despite this, group B showed moderate levels of disinhibition and apathy (i.e., greater than controls, but not as high as the other patient groups), as well as significant anterior temporal, OFC, and parietal lobe atrophy relative to controls. Compared with the other patient groups, however, group B did not show any regions of greater atrophy. Overall, this indicates that moderate symptoms of disinhibition and apathy may not necessarily impact on performance on the BART. Furthermore, this suggests that the version of the BART used in this study may not be as sensitive to everyday symptoms of disinhibition and apathy. While the CBI-R and DAS capture disinhibition and apathy symptoms in the context of daily life and quantify behaviour changes in relation to activities, relationships, and social norms (Radakovic et al., 2016; Wear et al., 2008), these behavioural changes may not necessarily be elicited in controlled, laboratory contexts, such as those during the BART. Nonetheless, this highlights the value of using a combination of questionnaire measures and cognitive tests in clinical practice to enable detailed assessment of symptom profiles across different contexts.

Conversely, group C popped fewer balloons and pumped less often than controls, but also collected less money, with a slightly slower reaction time. In terms of their informant-report measures, they showed the lowest disinhibition, relative to other patient groups, but some of the highest apathy scores. Their neuroimaging profile showed atrophy in the anterior and medial temporal lobes, as well as the insular cortex and OFC relative to controls, but relatively less atrophy compared with the other patient groups. This pattern of atrophy is largely consistent with previous research in apathetic dementia patients who were less motivated by rewards on a reward-learning task (Wong et al., 2023). Taken together, these results point to an apathetic profile of BART performance, whereby patients did not respond appropriately to maximise potential rewards. With regards to clinical management of this subgroup of patients, psychotherapeutic approaches, such as behavioural activation therapy, which seeks to foster positive reinforcement through engagement in pleasurable, productive, and personally meaningful activities (Richards et al., 2016), may be of value.

Finally, group D showed very similar performance to group C with the exception of mean reaction time, which was significantly slower than all other groups. In addition, group D had the lowest disinhibition and apathy on the informant-report measures compared with all other patient groups and controls. On neuroimaging, they showed predominantly posterior and hippocampal atrophy relative to the other patient groups, which was consistent with the higher proportion of AD patients in this group. In contrast to group C, however, group D’s poor BART performance appeared to be unrelated to disinhibition or apathy symptoms, but instead, driven by a high degree of caution, or greater overall cognitive impairment, which impacted their ability to respond in a timely manner. The latter explanation seems plausible, given that group D patients presented with the most severe cognitive impairment. As such, the clinical utility of the BART may be limited in this subgroup of patients.

Overall, the four clinical subgroups identified by our hierarchical cluster analysis largely map onto those reported by O’Connor et al. (2017), who categorised four phenotypic subgroups of bvFTD patients based on disinhibition and apathy symptoms reported on the CBI-R: those with primary severe disinhibition, primary severe apathy (similar to group C), severe disinhibition and apathy (similar to group A), and mild disinhibition and apathy (similar to group D). Given that those with severe apathy showed worse functional impairment and brain apathy (O’Connor et al., 2017), greater understanding of patients’ disinhibition and apathy symptom profiles from a combination of laboratory-based (e.g., BART) and informant-reported measures (e.g., CBI-R and DAS) may therefore provide more nuanced insights regarding prognosis and clinical management.

### Strengths, limitations, and future directions

Importantly, our hierarchical cluster analysis revealed a high degree of variability in BART performance within dementia subtypes and highlighted the value in considering multiple task outcome variables, as opposed to solely focusing on the mean adjusted pumps. The most salient example of this is the comparison of BART performance in group B, which performed very similarly to controls, versus group A, which showed the most “classically disinhibited” profile. While the two groups did not differ in terms of mean adjusted pumps, they showed vast differences in the number of balloons popped and total money collected. By focusing on the mean adjusted pumps only, as in previous BART studies (Lejuez et al., 2002, 2003; Strenziok et al., 2011), groups A and B would have appeared indistinguishable, thus overlooking important differences in risk-related affective decision-making.

Additionally, some methodological limitations warrant discussion. First, the Joggle Research version of the BART used in this study had a lower mean burst point (7) than the original BART (64) (Lejuez et al., 2002), which may have limited the range of responses. By limiting the burst point, however, a strength of this task version was the faster administration time (approximately 5 min), which enhanced its clinical utility. Nonetheless, future research is needed to examine the psychometric properties, particularly alternate-form reliability, of the BART version used in this study. As discussed above, another limitation of this study is that the participants did not earn real money on the BART, which may have impacted participants’ motivation. Indeed, the literature on this topic is mixed, with some evidence suggesting that financial incentives play a limited role in participants’ motivation in experimental studies (Read, 2005). Finally, some measures further distinguish between different dimensions of impulsivity and disinhibited behaviour (Grace & Malloy, 2001; Patton et al., 1995), which could not be disentangled in the current study using the CBI-R abnormal behaviour subscale. Future research should therefore incorporate more detailed measures, such as the Frontal Systems Behaviour Scale (Grace & Malloy, 2001) or the Barrett Impulsiveness Scale (Patton et al., 1995).

### Clinical and theoretical implications

From a clinical perspective, our results suggest that the BART has limited clinical utility in distinguishing between AD and bvFTD patients. Importantly, however, the within-diagnostic group variability in BART performance observed in our study underscores the utility of adopting a transdiagnostic approach for the assessment of affective decision-making deficits, compared to approaches that focus on clinical diagnosis only. While the AD patients in our sample presented with milder disinhibition and apathy symptoms as a group, relative to the bvFTD group, a proportion of these AD patients presented with more severe disinhibition/apathy symptoms and performed similarly to bvFTD patients on the BART. Likewise, a significant proportion of bvFTD patients performed similarly to controls on the BART and presented with milder disinhibition/apathy symptoms. A transdiagnostic approach, which focuses on assessing symptoms across patients with different dementia diagnoses, may provide crucial insights into potential shared neurocognitive mechanisms. In light of the co-occurrence of disinhibition and apathy symptoms, such knowledge may help inform the development of tailored, patient-centred interventions that target these overlapping symptoms.

From a theoretical standpoint, our findings highlight the importance of considering multiple outcome variables on affective decision-making tasks such as the BART, given the nuanced performance profiles this can reveal. Notably, this study was the first to explore the notion that maladaptive risk-related affective decision-making in dementia may be underpinned by both disinhibition and apathy symptoms. More research is needed to better understand how the co-existence of these symptoms impacts different aspects of affective decision-making, such as self-generated goal-directed behaviour and subjective valuation (Petitet et al., 2021).

### Conclusions

This study demonstrates the heterogeneous nature of affective decision-making deficits in dementia, using laboratory-based measures such as the BART. Our results provide the first empirical evidence that risky decision-making in bvFTD and AD is linked with symptoms of both disinhibition and apathy, as well as OFC and medial frontal atrophy. Greater understanding of these symptom profiles and underlying neurocognitive mechanisms may help to inform future development of transdiagnostic intervention approaches that target deficits in affective decision-making in dementia.

## Supplementary Information

Below is the link to the electronic supplementary material.

Supplementary file1 (DOCX 972 KB)

## Data Availability

The conditions of our ethics approval do not permit public archiving of anonymised study data. Readers seeking access to the data should contact the corresponding author. Access will be granted to named individuals in accordance with ethical procedures governing the reuse of clinical data, including completion of a formal data sharing agreement and approval of the local ethics committee. This study was not pre-registered.
