# Supplementary material for: Risky decision-making in dementia: Exploring neural correlates and related clinical symptoms
Source: Cogn Affect Behav Neurosci. 2025 Apr 11;25(5):1575–95. doi: 10.3758/s13415-025-01291-3 (PMC12464114; doi:10.3758/s13415-025-01291-3)
Supplement: Supplementary file 1 — Supplementary file1 (DOCX 972 KB) [file 13415_2025_1291_MOESM1_ESM.docx]

**Supplementary Materials**

**Supplementary Table 1** Partial correlations between BART variables and measures of apathy and disinhibition in bvFTD and AD patients, controlling for ACE-III scores

| BART performance variables | Disinhibition  (CBI-R abnormal behaviour subscale) | Apathy  (DAS) |
| --- | --- | --- |
| Total balloons popped | r(64) = .307, *p*=.021 | **r(63) = .454, *p*<.001** |
| Mean reaction time | **r(64) = -.398, *p*=.002** | **r(63) = -.451, *p*<.001** |
| Total money collected | r(64) = -.019, *p*=.889 | r(63) = -.229, *p=*.089 |
| Mean adjusted pumps | r(64) = .227, *p*=.093 | r(63) = .119, *p=*.381 |

*Note.* Statistically significant correlations in bold. Correlations are significant at *p*<.0125.

CBI-R = Cambridge Behavioural Inventory – Revised, DAS = Dimensional Apathy Scale. Degrees of freedom in brackets.

**Supplementary Table 2** Partial correlations between BART variables and measures of apathy and disinhibition in bvFTD and AD patients, controlling for CDR-FTLD scores

| BART performance variables | Disinhibition  (CBI-R abnormal behaviour subscale) | Apathy  (DAS) |
| --- | --- | --- |
| Total balloons popped | **r(64) = .319, *p*=.012** | **r(63) = .451, *p*<.001** |
| Mean reaction time | **r(64) = -.407, *p*=.001** | **r(63) = -.462, *p*<.001** |
| Total money collected | r(64) = .181, *p*=.163 | r(63) = -.059, *p=*.650 |
| Mean adjusted pumps | r(64) = .282, *p*=..027 | r(63) = .141, *p=*.279 |

*Note.* Statistically significant correlations in bold. Correlations are significant at *p*<.0125.

CBI-R = Cambridge Behavioural Inventory – Revised, DAS = Dimensional Apathy Scale. Degrees of freedom in brackets.

**Comparison of BART variables across groups identified through the hierarchical cluster analysis**

Group A comprised of 86% bvFTD patients and 14% AD patients. Group A patients popped the most balloons – significantly more than groups B, *p*<.001, C, *p*<.001, and D, *p*<.001. Group A patients also had the fastest reaction time, faster than groups C, *p*=.04, and D, *p*<.001, but not significantly different to group B, *p*=.99. Group A patients collected the lowest total money, significantly less money than groups B, *p*<.001, and D, *p*=.05, but not significantly different to group C, *p*=.18. Group A also had the highest mean adjusted pumps, significantly higher than group C, *p*<.001, but not groups B, *p*=.59, or D, *p*=.09.

Group B comprised of 58% bvFTD patients and 42% AD patients. Group B patients popped significantly more balloons than groups C, *p*<.001 and D, *p*=.01, but significantly less than group A, *p*<.001. Patients in this group had faster mean reaction times than group C, *p*=.02, and D, *p*<.001, but did not significantly differ from group A, *p*=.99. Group B patients collected the most total money, significantly more than groups C, *p*<.002, D, *p*=.02, and A, *p*<.001. Group B patients also had the highest mean adjusted pumps, significantly more than group C, *p*<.001, but not D, *p*=.08, or A, *p*=.59.

Group C comprised of 67% bvFTD patients and 33% AD patients. Group C patients popped significantly fewer balloons than groups B, *p*<.001, and A, *p*<.001, had a slower mean reaction time than groups B, *p*=.02, and A, *p*=.04, collected significantly less money than group B, *p*<.001, but did not differ from group A, *p*=.18 in terms of money collected. Group C patients’ mean adjusted pumps were significantly lower than group B, *p*<.001, group D, *p*=.04, and group A, *p*<.001.

Group D comprised of 33% bvFTD patients and 67% AD patients. This group performed similarly to group C on balloons popped, *p*=.42 and total money collected, *p*=.38. However, group D patients’ mean reaction time was the slowest of all the groups, significantly slower than groups B, *p*<.001, C, *p*<.001, and A, *p*<.001. Group D also had higher mean adjusted pumps than group C, *p*=.04, but not significantly different from groups B, *p*=.08, or A, *p*=.09.

The control group popped more balloons than groups C, *p*<.001, and D, *p*=.01, but less than group A, *p*=.02, and no significant difference to group B, *p*=.87. Mean reaction time is the only BART variable in which the controls differed from group B, *p*=.04, with controls having a faster reaction time than groups B, C, *p*<.001, and D, *p*<.001, but no significant difference to group A, *p*=.10. Controls collected more money than groups C, *p*<.001, D, *p*=.01, and A, *p*<.001, but not B, *p*=.86. Controls had more mean adjusted pumps than group C, *p*<.001, but not B, D, or A, *p*-values > .14.

**Supplementary Table 3.** *Demographic, BART outcome, disinhibition and apathy variables across controls and groups A-D identified through the hierarchical cluster analysis.*

| **Variables** |  |  | | ***p*-values** | | | | | | | | | |  |  |  |  |  |
| --- | --- | --- | --- | --- | --- | --- | --- | --- | --- | --- | --- | --- | --- | --- | --- | --- | --- | --- |
|  | **A** | **B** | **C** | | **D** | **Con** | **Group differences** | **A v B** | **A v C** | **A v D** | **B v C** | **B v D** | **C v D** | | **Con v A** | **Con v B** | **Con v C** | **Con v D** |
| **Age** | 63.30 (6.86) | 61.85 (8.27) | 67.23 (8.51) | | 63.74 (6.28) | 66.75 (5.67) | *F*(4)=2.92, *p*=.024 | .99 | .93 | .99 | .15 | .99 | .95 | | .94 | .03 | .99 | .95 |
| **Education (years)** | 12.29 (1.60) | 13.01 (2.74) | 13.02 (2.91) | | 14.61 (4.11) | 14.46 (3.04) | *F*(4)=8.38, *p*=.08 |  |  |  |  |  |  | |  |  |  |  |
| **Disease duration (years)** | 3.69 (1.82) | 5.37  (2.76) | 3.70 (2.17) | | 4.83 (4.51) | - | *F*(3)=5.04, *p*=.17 |  |  |  |  |  |  | |  |  |  |  |
| **CDR-FTLD** | 7.64 (3.47) | 5.22 (1.85) | 3.85 (1.85) | | 5.56 (4.14) | 0.32 (0.52) | *F*(4)=78.54, *p*<.001 | .33 | .12 | .26 | .32 | .65 | .73 | | <.001 | <.001 | <.001 | <.001 |
| **ACE-III** | 72.00 (13.81) | 74.81 (11.30) | 77.00 (16.38) | | 49.00 (20.47) | 94.81 (3.64) | *F*(4)=68.03, *p*<.001 | .77 | .48 | .24 | .51 | .06 | .04 | | <.001 | <.001 | <.001 | <.001 |
| **Balloons popped** | 23.43  (5.83) | 12.24  (2.82) | 7.00  (2.33) | | 7.56  (5.10) | 11.98  (3.43) |  | <.001 | <.001 | <.001 | <.001 | 0.01 | 0.42 | | 0.01 | 0.87 | <.001 | 0.01 |
| **Mean reaction time** | 869.71  (459.03) | 1028.06  (421.96) | 1411.96  (445.91) | | 3621.59  (558.25) | 759.26  (313.45) |  | 0.99 | 0.04 | <.001 | 0.02 | <.001 | <.001 | | 0.99 | 0.04 | <.001 | <.001 |
| **Total money collected** | 30.43  (21.63) | 100.84  (14.71) | 69.80  (22.96) | | 76.44  (33.83) | 99.67  (18.57) |  | <.001 | 0.18 | 0.05 | <.001 | 0.02 | 0.38 | | <.001 | 0.86 | <.001 | 0.01 |
| **Mean adjusted pumps** | 5.40  (3.92) | 5.03  (1.11) | 2.23  (0.81) | | 3.70  (2.20) | 4.8726  (1.47) |  | 0.586 | <.001 | 0.085 | <.001 | 0.082 | 0.036 | | 0.43 | 0.662 | <.001 | 0.14 |
| **CBI-R abnormal behaviour** | 42.26  (13.70) | 37.05  (22.35) | 16.99  (13.23) | | 16.67  (19.54) | 3.96  (4.93) |  | 0.52 | 0.03 | 0.01 | 0.02 | 0.01 | 0.60 | | <.001 | <.001 | 0.01 | 0.07 |
| **DAS** | 56.17  (9.75) | 48.50  (9.89) | 48.00  (10.14) | | 39.56  (9.25) | 17.89  (9.76) |  | 0.56 | 0.65 | 0.02 | 0.99 | 0.15 | 0.42 | | <.001 | <.001 | <.001 | <.001 |

*Note.* C: controls, M: mean, SD: standard deviation, CDR-FTLD: Clinical Dementia Rating Scale – Frontotemporal Lobar Degeneration, ACE-III: Addenbrooke’s Cognitive Examination, 3^rd^ edition, CBI-R: Cambridge Behavioural Inventory, DAS: Dimensional Apathy Scale.

Values in square brackets indicate maximum scores.

**Diagnosis group differences in grey matter atrophy**

**AD vs controls**

Relative to controls, AD patients showed lower grey matter intensity in the bilateral medial temporal lobe, extending to the medial parietal and temporooccipital cortices bilaterally.

**bvFTD vs controls**

Relative to controls, bvFTD patients showed lower grey matter intensity in the medial temporal lobe, extending to the medial parietal lobe, the basal ganglia, medial frontal cortex, and orbitofrontal cortex, bilaterally.

**AD vs bvFTD**

AD patients showed lower grey matter intensity than bvFTD patients in the precuneous cortex, extending to the intracalcarine and supracalcarine cortex, the postcentral gyrus, and the superior lateral occipital cortex.

**bvFTD vs AD**

bvFTD patients had lower grey atter intensity than AD patients in the orbitofrontal cortex, extending to the frontal pole, inferior frontal gyrus, pars triangularis, frontal operculum cortex and central operculum cortex, and back to the insular cortex and temporal pole.

**Supplementary Table 4.** Regions of significant grey matter atrophy across diagnosis groups

|  |  | MNI coordinates | | |  |
| --- | --- | --- | --- | --- | --- |
| Regions | Hemisphere | X | Y | Z | Cluster size |
| **AD vs controls** |  |  |  |  |  |
| Temporal pole; insular cortex; anterior superior temporal gyrus; middle temporal gyrus; anterior inferior temporal gyrus; parahippocampal gyrus; anterior temporal fusiform cortex; temporooccipital middle and inferior temporal gyri; temporooccipital fusiform cortex; occipital fusiform gyrus; inferior lateral occipital cortex; occipital pole | Left | -32 | 6 | -50 | 74,668 |
| Frontal pole | Left | -16 | 64 | -2 | 644 |
| Frontal pole; superior frontal gyrus | Right | 10 | 48 | 34 | 51 |
| **bvFTD vs controls** |  |  |  |  |  |
| Anterior temporal fusiform cortex; anterior temporal parahippocampal cortex; temporal pole; anterior superior temporal gyrus; posterior middle temporal gyrus; planum polare; central operculum cortex; insular cortex; subcallosal cortex; paracingulate gyrus; caudate; putamen; accumbens; precentral gyrus; inferior frontal gyrus; middle frontal gyrus; frontal pole; orbitofrontal cortex | Bilateral | 30 | -6 | -52 | 78,441 |
| **AD vs bvFTD** |  |  |  |  |  |
| Precuenous cortex; intracalcarine cortex; supracalcarine cortex; postcentral gyrus; superior lateral occipital cortex | Bilateral | 12 | -62 | 18 | 18,663 |
| **bvFTD vs AD** |  |  |  |  |  |
| Orbitofrontal cortex; frontal pole; inferior frontal gyrus; pars triangularis; frontal operculum cortex; central operculum cortex; insular cortex; temporal pole | Right | 40 | 18 | -14 | 7659 |
|  |  |  |  |  |  |

**Supplementary Figure 1.** Regions of significant grey matter atrophy in A) AD patients vs. controls and B) bvFTD patients vs. controls.

**
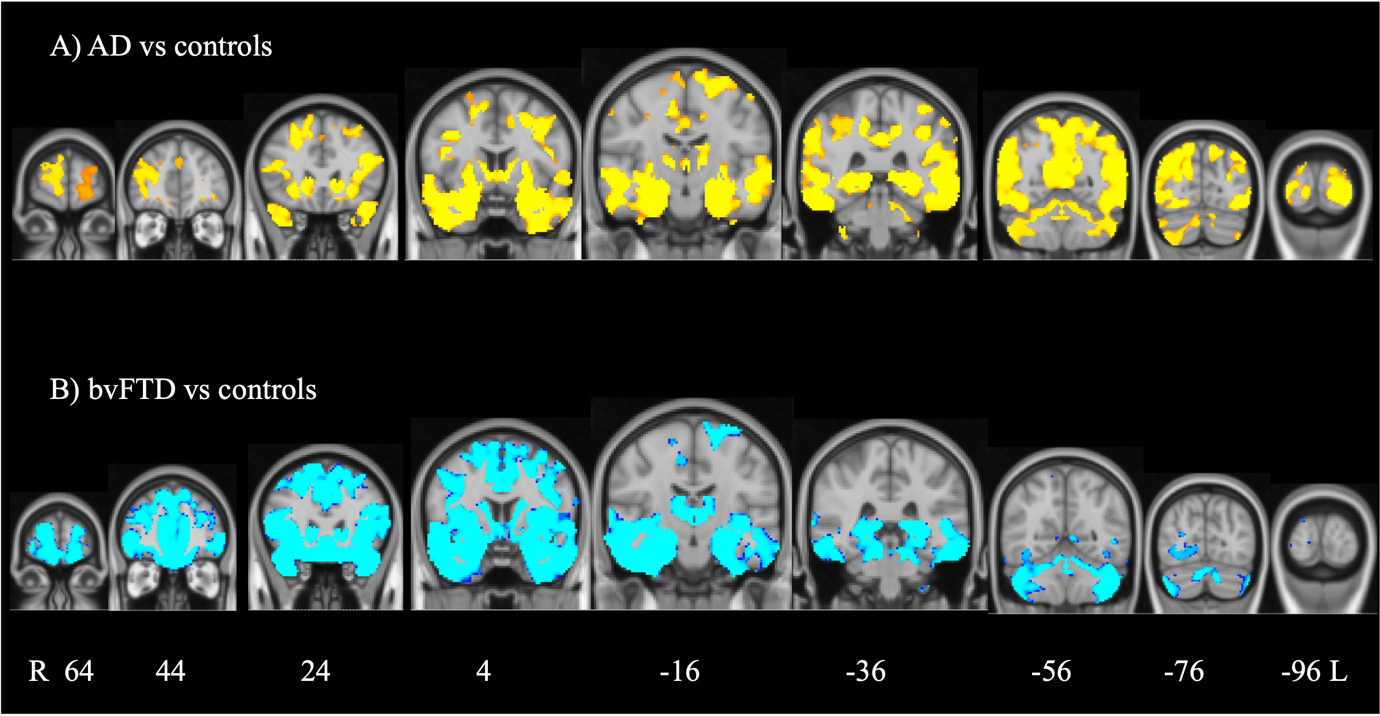
**

*Note.* Results are family-wise error corrected and statistically significant at *p*<.05.

**Supplementary Figure 2.** Regions of significant grey matter atrophy in A) AD vs. bvFTD patients and B) bvFTD vs. AD patients

**
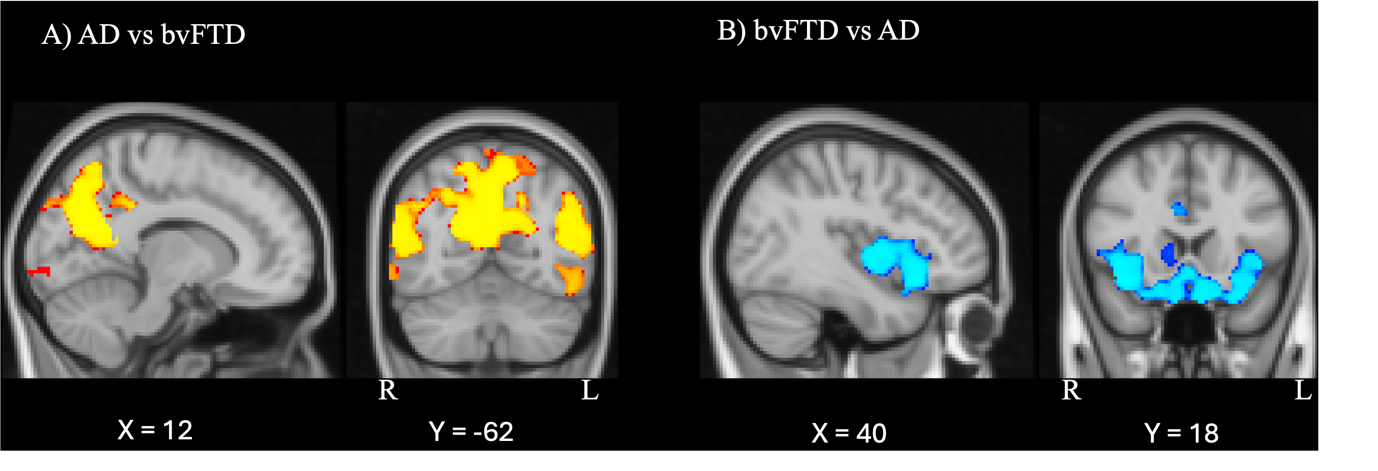
**

*Note.* Results are family-wise error corrected and statistically significant at *p*<.05.
